# Endoscopic variceal ligation combined with sclerotherapy for management of gastroesophageal variceal bleeding in pediatric patients: a single-center retrospective study

**DOI:** 10.3389/fped.2024.1325419

**Published:** 2024-07-01

**Authors:** Ming-Ming Li, Fang Sun, Man-Xiu Huai, Chun-Ying Qu, Feng Shen, Yi Zhang, Lei-Ming Xu

**Affiliations:** Department of Gastroenterology, Xinhua Hospital, Shanghai Jiaotong University School of Medicine, Shanghai, China

**Keywords:** gastroesophageal variceal bleeding, portal hypertension, endoscopic sclerotherapy, endoscopic ligation, secondary prophylaxis

## Abstract

**Objectives:**

Portal hypertension (PH) frequently gives rise to severe and life-threatening complications, including hemorrhage accompanied by the rupture of esophageal and gastric varices. In contrast to the guidelines for the management of PH in adults, the optimal endoscopic management of variceal bleeding for secondary prophylaxis in children remains unclear. The present study evaluated the efficacy and safety of endoscopic variceal ligation (EVL) and endoscopic sclerotherapy (EST) to control gastroesophageal variceal bleeding in children.

**Methods:**

This retrospective study included children with gastroesophageal variceal bleeding who underwent EST or EVL at Xinhua Hospital, Shanghai Jiaotong University School of Medicine, between February 2013 and March 2020. Short-term hemostasis rate and long-term rebleeding rate were evaluated. Adverse events related to the procedures, such as esophageal ulcer, esophageal stricture, abnormal embolization, pneumonia and perforation, were also recorded.

**Results:**

EVL (*n* = 8) and EST (*n* = 13) were performed successfully in all pediatric patients diagnosed with moderate to severe esophageal varices concurrent with gastric varices. Hemostasis was achieved during episodes of upper gastrointestinal bleeding. The mean volume of each single aliquot of cyanoacrylate injected was 0.3 ± 0.1 ml (range: 0.1–0.5 ml). Varices were eradicated in six (75%) of the eight patients who underwent EVL after a median 2 (range: 1–4) procedures and a median time of 3.40 months (range: 1.10–13.33 months). Eleven (52.4%) of the 21 patients developed rebleeding events, with the mean duration of hemostasis being 11.1 ± 11.6 months (range 1.0–39.2 months). No treatment-related complications, for example, distal embolism, occurred except for abdominal pain in one patient (4.8%).

**Conclusions:**

EST, alone or in combination with EVL, is an effective and safe method of managing gastroesophageal variceal hemorrhage in children undergoing secondary prophylaxis.

## 1 Introduction

Portal hypertension (PH) is a frequent complication of chronic liver disease in adults. PH in pediatric patients, however, is usually caused by portal vein obstruction, due to conditions such as biliary atresia, congenital hepatic fibrosis, portal cavernous formation and hepatic vein obstruction. Gastroesophageal varices secondary to PH in children can give rise to massive upper gastrointestinal hemorrhage with mortality rates ranging from 5% to 19% (1, 2). Treatment options in adults include surgical procedures, endoscopic minimally invasive methods and β-blockers; however, the optimal treatment of gastroesophageal varices in pediatric patients remains unclear (3).

The prevention of a sentinel variceal bleed by endoscopic procedures can improve patient survival. Endoscopic procedures for the treatment of variceal hemorrhage include endoscopic variceal ligation (EVL) and endoscopic sclerotherapy (EST). The latter therapy requires a sclerosing agent with or without a tissue adhesive agent. These treatments of adult patients are dependent on patient condition. Few studies to date, however, have assessed the management of PH in children, with clinical practice varying widely among physicians. These variations suggest that many pediatric patients receive suboptimal treatment and indicate the need for guidelines to manage PH in children. The present study describes the endoscopic treatment of PH in children at Xinhua Hospital, Shanghai Jiaotong University School of Medicine, between February 2013 and March 2020.

## 2 Patients and methods

### 2.1 Study design

This retrospective observational study included pediatric patients who underwent endoscopic treatment for PH at Xinhua Hospital, Shanghai Jiaotong University School of Medicine, between February 2013 and March 2020. The study was performed in accordance with the Helsinki Declaration and was approved by the Ethics Committee of Shanghai Jiaotong University School of Medicine. Written informed consent was obtained from all patients and their legal guardian(s), who had been advised of the potential risks before the endoscopic procedure.

### 2.2 Patients

The present study retrospectively enrolled consecutive pediatric patients (age <12 years) with variceal bleeding, manifesting as hematemesis or melena, who underwent EVL or EST at Xinhua Hospital between February 2013 and March 2020. The pre- and post-procedural clinical characteristics of these patients were collected and analyzed.

### 2.3 Endoscopic definitions

Esophageal and gastric varices can be divided into four types based on their characteristics and locations in the stomach (4). Type 1 gastroesophageal varices (GOV1), the most common type, are defined as continuous esophageal varices extending into the stomach below the cardia and along the lesser curvature; and type 2 gastroesophageal varices (GOV2) have been described as continuations of esophageal varices extending into fundus along the greater curvature of the stomach beyond the cardia. In addition, type 1 isolated gastric varices (IGV1) have been defined as isolated gastric varices, mostly located at the gastric fundus, and type 2 isolated gastric varices (IGV2) have been defined as isolated gastric varices located around the gastric body, antrum or pylorus (5).

Esophageal varices are graded according to their shapes and the presence or absence of red sign (6). Grade Ⅰ esophageal varices are linear or slightly tortuous without a red sign. Grade Ⅱ esophageal varices are linear or slightly tortuous with a red sign; or show snakelike tortuous uplift but no red sign. Grade Ⅲ esophageal varices have a serpentine tortuous uplift with a red sign or are beaded, nodular or tumorous with or without a red sign.

### 2.4 Details of endoscopic management for esophagogastric variceal bleeding (EGVB)

Endoscopic procedures were performed by two experienced endoscopists using a forward-viewing electronic gastroscope (Olympus GIF-XQ290), with the patient under general anesthesia with endotracheal intubation.

**Figure 1:**
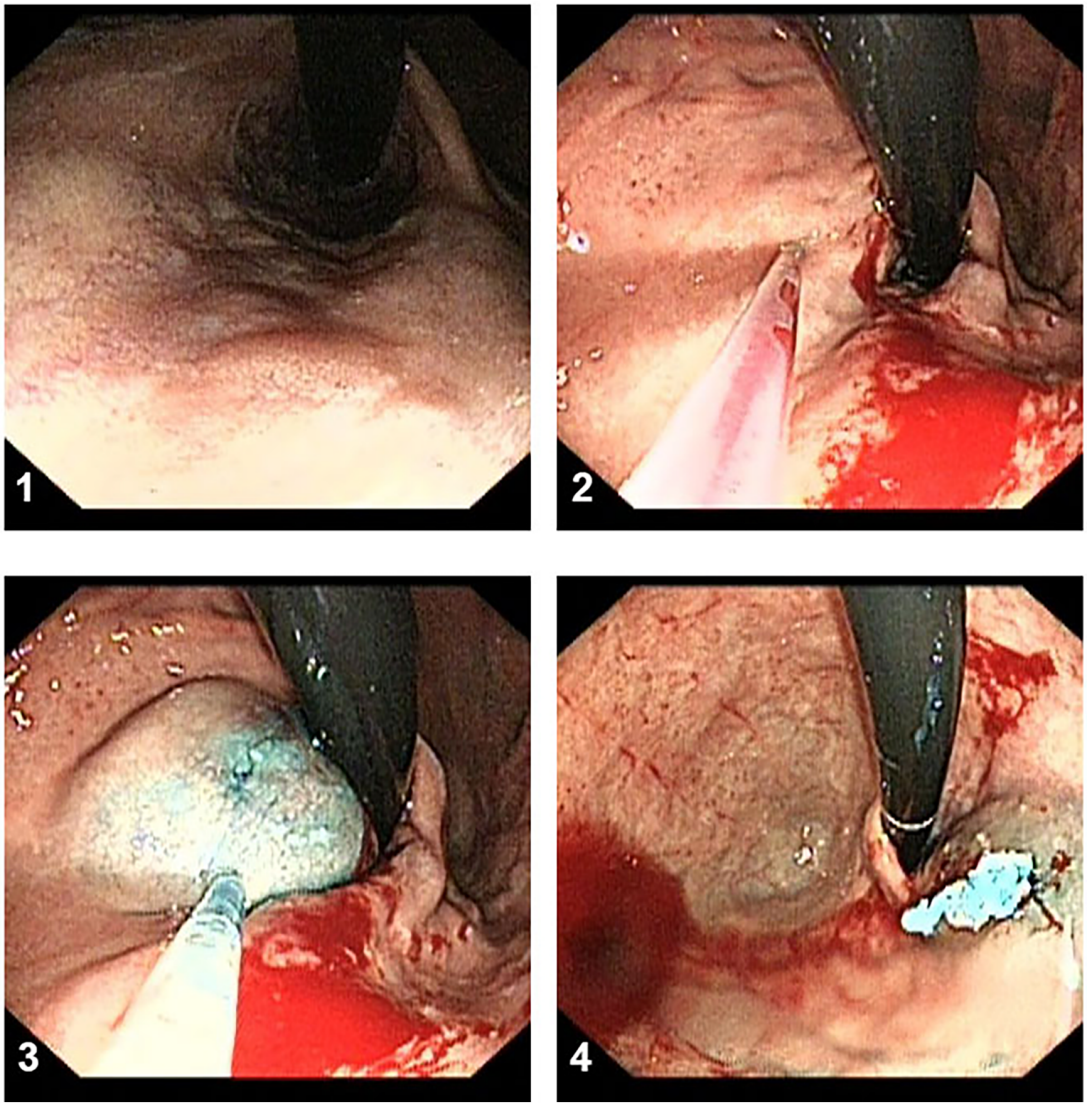
The procedure of EST treatment. (**1**) Gastric varices were located at the fundus of gastric. (**2**) The transparent teflon injector was injected into the varices and blood could be seen. (**3**) A tissue adhesive agent between two injections of sclerosing agent was injected into the varices. (**4**) The procedure was successfully conducted.

EST procedure as shown in Figure 1, EST was based on the use of a sclerosing agent and a tissue adhesive agent. Patients with gastric varices were managed with a “sandwich method” by the injection of a tissue adhesive agent (N-butyl-α-cyanoacrylate, 0.5 ml each, Beijing Fu'aile Technology Development Co., Ltd., China) between two injections of sclerosing agent (3% polidocanol, 1 ml/piece, Shaanxi Tianyu Pharmaceutical Co., Ltd., China). The external sheath tube of the 23-gauge transparent teflon injector was gently pressed on the injection point for 3–5 s before pulling out the needle to prevent overflow of the tissue adhesive. Depending on the diameter of each variceal vein, 0.5–2 ml tissue adhesive was required for gastric varices. Severe gastric varices required injections at two or more sites to avoid ectopic embolism of a large amount of tissue adhesive.

Esophageal varices were injected intravariceally with the sclerosing agent 3% polidocanol and the staining agent methylene blue, without the use of tissue adhesive agent. Methylene blue could predict the trend of sclerosing agent. Generally 0.5–1.5 ml sclerosant was injected per puncture, based on the size of the varix, for a total of 6–10 ml. Each variceal column received one to three injections, starting just above the gastroesophageal junction and proximally at 2-cm intervals. Patients received sclerotherapy every 21 days until the varices were obliterated completely and no variceal column was seen.

**Figure 2:**
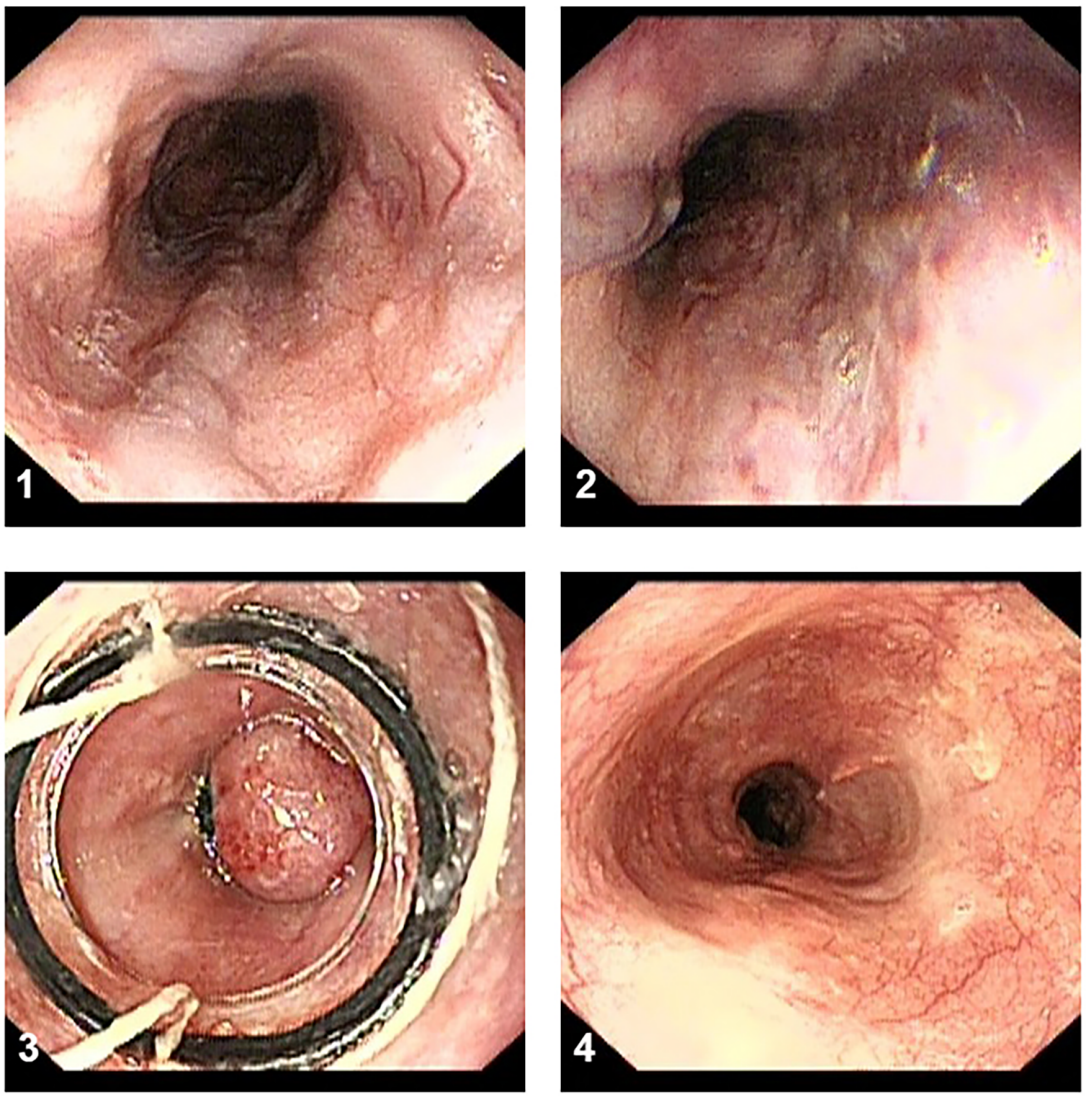
The procedure of EVL treatment. (**1,2**) The esophageal varices have a serpentine tortuous uplift with a red sign. (**3**) The variceal cord was ligated using two bands. (**4**) The surveillance endoscopy showed the white scars after 3 months.

EVL procedure EVL was performed with five or six multiband ligator devices (Cook Medical, Limerick, Ireland), each consisting of a transparent “Opti-Vu” barrel with preloaded latex rubber bands and an attached trigger cord, multiband ligator handle, and loading catheter. Ligation was started at or just proximal to the esophagogastric junction and extended toward the head in a slightly spiral fashion within 5 cm distal to the esophagus on all visible varices. During each session, each variceal cord was ligated using one or two bands. EVL was performed every 2–8 weeks until varices were eradicated, followed by surveillance endoscopy 3–6 months after variceal eradication, and every 6–12 months thereafter. The procedure was shown in Figure 2.

### 2.5 Evaluation of therapeutic outcomes

Control of active bleeding was defined as complete cessation of bleeding with stable vital signs for more than 48 consecutive hours after endoscopic treatment. Variceal eradication was defined as the obliteration of all visible varices or their reduction to tiny thrombosed remnants or a grade Ⅰ size that can no longer be suctioned into the ligating device. Successful hemostasis of active gastric variceal bleeding was defined, according to the Baveno V criteria, as an absence of upper gastrointestinal bleeding for the first 120 h after cyanoacrylate injection. Unsuccessful hemostasis was defined as death or the need to change the therapy due to (1) fresh hematemesis or naso-gastric aspiration that removed ≥100 ml of fresh blood ≥2 h after the procedure; (2) the development of hypovolemic shock; or (3) a 3 g/L drop in hemoglobin within any 24 h period in the absence of transfusions. Rebleeding was defined as unsuccessful secondary prophylaxis, clinically significant rebleeding from PH sources after day 5, or recurrent melena or hematemesis resulting in (1) hospital admission; (2) blood transfusion; (3) a 3 g/L drop in hemoglobin concentration; or (4) death within 6 weeks. Treatment failure was defined as: (1) a failure to control index active bleeding or a rebleeding episode following two separate attempts of the same endoscopic treatment, (2) three or more rebleeding episodes that required endoscopic treatment and transfusion, (3) death related to rebleeding or complication, and (4) a physician's decision to change treatment modality. Major complications were defined as any adverse event (e.g., complicated esophageal ulcer, esophageal stricture, abnormal embolization, pneumonia, pleural effusion, or perforation) that required hospitalization. A complicated esophageal ulcer was defined as a treatment-induced esophageal ulcer that was associated with bleeding or significant dysphagia, or led to the postponement of scheduled endoscopy (7–11).

### 2.6 Post-procedural care and follow-up

Patients were fasted for 24 h after endoscopic treatment, followed by a liquid diet for 3 days. Vital signs were closely monitored, as were indications of bleeding, abdominal pain, chest pain, and dysphagia. Patients were administered necessary post-procedural medications for portal pressure reduction, acid suppression, infection prevention and nutritional support. The need for endoscopic retreatment was based on the improvement of varicose veins.

## 3 Results

### 3.1 Clinical presentation and laboratory features

The 21 pediatric patients with concomitant gastroesophageal varices who received endoscopic hemostasis included 10 boys and 11 girls, of median age 5.5 ± 3.0 years (range: 1–11 years), including six patients aged <3 years (Table 1). The etiology of PH included cavernous degeneration of the portal vein (CTPV), biliary atresia, congenital hepatic fibrosis, Caroli's disease, and Niemann-pick's disease. According to the Sarin classification, 20 patients (95.2%) were categorized as having type 1 gastroesophageal varices (GOV1), which was much more than IGV1 (only 4.8%). Evaluation of severity showed that 18 (85.7%) children had grade III and three (14.3%) had grade II esophageal varices. The mean pediatric end-stage liver disease (PELD) score for patients younger than 12 years was −7.2 ± 4.69. The average platelet count was 125.1 ± 96.5/ml, which could be regarded as a noninvasive indicator of esophageal varices in children with PH. Six (28.6%) patients underwent surgery to relieve PH prior to endoscopic treatment, including the Kasai procedure or portojejunal anastomosis, splenectomy, liver transplant, and Rex shunt.

**Table 1:** Clinical characteristics of the 21 pediatric patients.

Clinical characteristics	*N* = 21
Male/female	10/11
Age (years)	
Mean ± SD	5.5 ± 3.0
Range	1–11
Etiology	
Cavernous degeneration of portal vein (CTPV)	17 (81.0%)
Biliary atresia	1 (4.8%)
Congenital hepatic fibrosis	1 (4.8%)
Caroli's disease	1 (4.8%)
Niemann-Pick's disease	1 (4.8%)
Sarin classification of the gastroesophageal varices	
GOV1	20 (95.2%)
IGV1	1 (4.8%)
Grade of the esophageal varices	
Grade Ⅱ	3 (14.3%)
Grade Ⅲ	18 (85.7%)
Platelet counts, mean ± SD (/ml)	125.1 ± 96.5
PELD score (<12 years), mean ± SD	−7.2 ± 4.69
Related-surgery history	6/21 (28.6%)

Data are expressed as mean ± SD or as number (%).

PELD, pediatric end-stage liver disease.

### 3.2 Outcomes and complications

**Figure 3:**
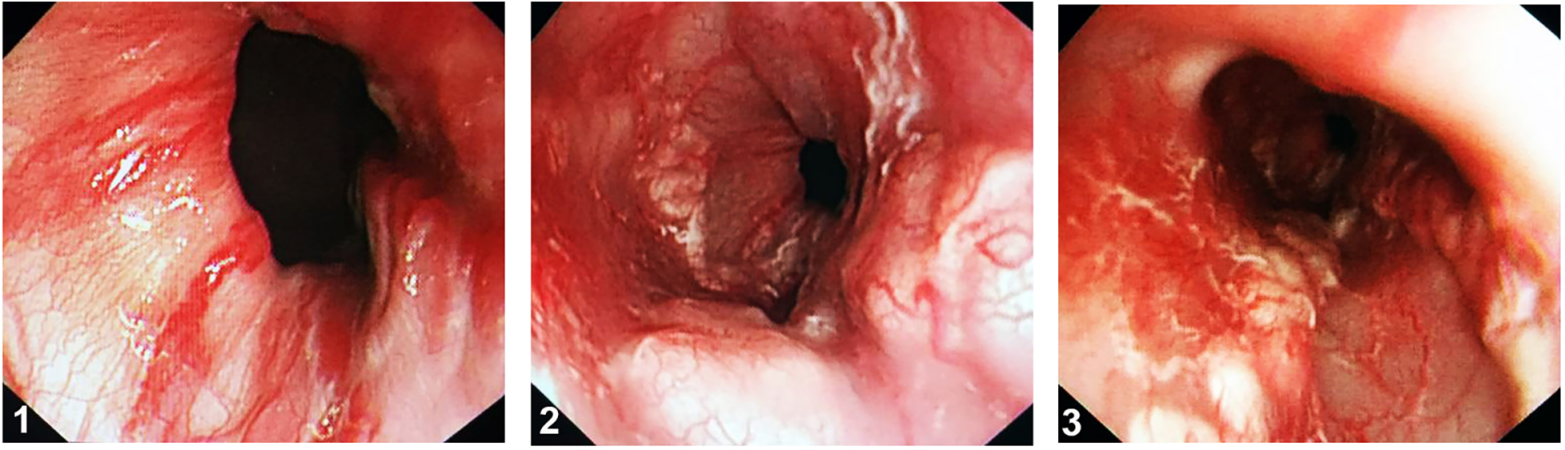
Active gastric variceal bleeding. (**1**) Varices have a serpentine tortuous uplift with a red sign. (**2,3**) Visible bleeding or clotted blood over a esophagogastric varix.

Outcomes and complications of endoscopic treatment are summarized in Tables 2, 3. Diagnostic esophagogastroscopy within 24 h confirmed that all patients had active gastric variceal bleeding (visible bleeding or clotted blood over a gastric varix, as shown in Figure 3), in addition to the presence of large gastric varices and other sources of bleeding that often accompany with hematemesis and melena. All 21 patients underwent EST (*n* = 13) or EVL (*n* = 8) for esophageal varices, including sequential application of sclerosant and tissue adhesive for gastric varices as secondary prophylaxis. Of the 21 patients who underwent endoscopic management, 13 (61.9%) showed primary eradication of varices after a mean 1.4 ± 0.8 sessions. The mean volumes of single aliquots of polidocanol and cyanoacrylate required to obliterate the concomitant gastric varices were 9.1 ± 1.2 ml and 0.6 ± 0.1 ml, respectively. The mean number of injection points for each session was 1.7 ± 0.7. The successful hemostasis rate for upper gastrointestinal bleeding was as high 100%, as demonstrated by the increase of hemoglobin and the absence of fecal occult blood. During the follow-up period of 2.6–90.4 months, eight (38.1%) of the 21 patients with recurrent rebleeding required endoscopic management, with three (14.3%) of the 21 patients developing rebleeding within 12 months.

**Table 2:** Details of endoscopic therapy.

Treatment details	*N* = 21
Esophageal varices	
EST	13 (61.9%)
EVL	8 (38.1%)
Gastric varices	
Mean single aliquot of polidocanol (ml)	9.1 ± 1.2
Mean single aliquot of cyanoacrylate (ml)	0.6 ± 0.1
Mean injection points per session	1.7 ± 0.7
Mean number of sessions	1.4 ± 0.8

**Table 3:** Treatment outcomes and complications.

Treatment outcome	*N* = 21
Primary varix eradication	13/21 (61.9%)
Hemostasis	21/21 (100%)
Follow-up duration (months)	2.6–90.4
Duration of hemostasis (months)	33.4 ± 27.6
Rebleeding (%)	8/21 (38.1%)
Rebleeding within 12 months	3/21 (14.3%)
Complications	2/21 (9.5%)

Data are expressed as mean ± SD or as number (%).

None of these patients developed acute endoscopy-related complications during endoscopic therapy. Two patients (9.5%) subsequently developed treatment-related complications, with one each developing esophageal stricture and esophageal ulcer, requiring extended hospitalization. Other complications, such as treatment-associated infection, perforation, and distant emboli, did not occur. One 10-year-old patient required repeated endoscopic stent placement and endoscopic balloon dilatation due to esophageal stricture, but subsequently recovered.

## 4 Discussion

Variceal bleeding, a major complication of PH secondary to decompensated cirrhosis or portal vein obstruction, is associated with significant mortality rates in adults. Mortality rates in children with a first variceal bleed, however, are relatively low, partly because the underlying liver disease may be well-compensated at the onset of bleeding. Furthermore, comorbidities typically seen in adults are usually absent in children, perhaps because the etiology of PH differs markedly in children and adults. Children have extrahepatic PH with normal liver function, whereas most adults have hepatic fibrosis and cirrhosis with severe jaundice or ascites (9, 10). Most of the pediatric patients in the present study were found to have well-preserved liver function, as PH in 17 (81%) of these patients was indicative of cavernous degeneration of the portal vein due to unexplained primary causes.

However, most of these children had simultaneous esophageal and gastric varices. Grade 3 varices and grade 2 varices with esophageal reddish spots have been reported to be independent risk factors for EGVB (3, 12–14). The persistence of chronic pathological processes and the continual progress of portal pressure make it crucial to identify methods that can effectively control acute bleeding and prevent rebleeding due to EGVB. Endoscopic treatment has become a mainstay of long-term management of EGVB, significantly reducing mortality rates in adults. In contrast, optimal endoscopic treatment for pediatric patients has not been determined.

EVL has been shown to be effective and safe as first-line treatment to prevent or control esophageal variceal bleeding in both adults and children (5, 15). Compared with EST, EVL is thought to bring about variceal eradication after fewer sessions and with a lower recurrence rate. EVL, however, is often unsuitable for small children because conventional banding devices have not yet been developed for the thin esophageal lumen of children, potentiating esophageal injury from overtubes (10). Moreover, the angled position of the fundus in children may prevent the device tip from reaching the bleeding loci. EVL may be contraindicated due to the formation of an esophageal scar or the risk of mild varicosity. EVL is also difficult to perform in patients with reticular communicating vessels and with post-treatment residual varix scarring.

The Baveno V consensus has recommended use of a tissue adhesive agent, such as N-butyl-2-cyanoacrylate (histoacryl), as first-line treatments for endoscopic variceal obliteration of gastroesophageal varices and isolated gastric varices in adults (10). EST occludes the veins by destroying endothelial cells and producing aseptic chemical inflammation, which may subsequently result in fibrous hyperplasia of the esophageal walls. In this study, the mean volumes of single aliquots of cyanoacrylate required to obliterate the concomitant gastric varices were 0.6 ± 0.1 ml, and the mean number of injection points for each session was 1.7 ± 0.7. In summary, it is necessary to target the injection of cyanoacrylate into the varicose vein. Endoscopists could determine the injection volume based on the length or the diameter of the target vein, such as veins with diameter of 1 cm required injection of 1 ml of cyanoacrylate. Moreover, the following are some tips for endoscopic management provided below. The low rate of post-procedural ulcer after intravenous injection may benefit patients due to the administration of tissue adhesive and the temporary oppression before removing the needle. The decreased overflow of tissue adhesive can obliterate the varix and prevent necrosis of the local mucosa.

Despite endoscopic variceal obliteration with cyanoacrylate being more applicable to small children, caution is needed prior to its use for gastric varices in children. Fatal complications, such as systemic embolism, have been reported in patients treated with cyanoacrylate for variceal bleeding (16). Many articles reported pulmonary, splenic, and even pancreatic embolisms after EST (17–21). Although there were no cases of embolism in the study, we cannot ignore them. Splenic embolism is the most common complication, and it is always caused by excessive injection of cyanoacrylate, leading to the emboli reaching the splenic vein through collateral circulation. When the complication occurs, symptoms such as fever and abdominal distension may appear. Abdominal ultrasound or CT scans could detect and confirm the splenic embolism. Fortunately, most patients could recover after conservative treatment. We recommend that the dose of cyanoacrylate injected at each point during the treatment should not exceed 1–2 ml, and the total amount injected should not exceed 4 ml per session. Moreover, the endoscopic procedure of injection should be administered quickly. It could effectively reduce the incidence of embolisms after EST management.

Because the principal problem of persistently progressive portal hypertension cannot be easily solved, long-term and repeated endoscopic surveillance and effective management are necessary to prevent recurrent variceal bleeding. However, this study was limited by its inclusion of a small number of patients. Large, multicenter randomized clinical trials are required to determine the efficacy and safety of tissue adhesives, such as N-butyl-2-cyanoacrylate and 2-octyl-cyanoacrylate, for acute gastric variceal bleeding. Few studies to date have evaluated methods to manage PH in children, and clinical practice varies widely among physicians, suggesting that many patients may receive suboptimal care.

## 5 Conclusions

This study suggests that endoscopic vascular obliteration using EVL or EST may be effective in children for initial hemostasis of bleeding due to concomitant gastroesophageal varices, with a low rate of adverse events.

## Data Availability

The raw data supporting the conclusions of this article will be made available by the authors, without undue reservation.

## References

[1] Ling SC Walters T McKiernan PJ Schwarz KB Garcia-Tsao G Shneider BL. Primary prophylaxis of variceal hemorrhage in children with portal hypertension: a framework for future research. J Pediatr Gastroenterol Nutr. (2011) 52(3):254–61. 10.1097/MPG.0b013e318205993a 21336158 PMC3728696

[2] Maksoud-Filho JG Gonçalves ME Cardoso SR Gibelli NE Tannuri U. Long-term follow-up of children with extrahepatic portal vein obstruction: impact of an endoscopic sclerotherapy program on bleeding episodes, hepatic function, hypersplenism, and mortality. J Pediatr Surg. (2009) 44(10):1877–83. 10.1016/j.jpedsurg.2009.02.074 19853741

[3] Shneider BL de Ville de Goyet J Leung DH Srivastava A Ling SC Duché M Primary prophylaxis of variceal bleeding in children and the role of MesoRex bypass: summary of the Baveno VI pediatric satellite symposium. Hepatology. (2016) 63(4):1368–80. 10.1002/hep.28153 26358549

[4] Bandika VL Goddard EA De Lacey RD Brown RA. Endoscopic injection sclerotherapy for bleeding varices in children with intrahepatic and extrahepatic portal venous obstruction: benefit of injection tract embolisation. S Afr Med J. (2012) 102(11 Pt 2):884–7. 10.7196/samj.6263 23116751

[5] Zargar SA Javid G Khan BA Yattoo GN Shah AH Gulzar GM Endoscopic ligation compared with sclerotherapy for bleeding esophageal varices in children with extrahepatic portal venous obstruction. Hepatology. (2002) 36(3):666–72. 10.1053/jhep.2002.35278 12198659

[6] Duché M Ducot B Tournay E Fabre M Cohen J Jacquemin E Prognostic value of endoscopy in children with biliary atresia at risk for early development of varices and bleeding. Gastroenterology. (2010) 139(6):1952–60. 10.1053/j.gastro.2010.07.004 20637201

[7] Dilawari JB Chawla YK Ramesh GN Mitra SK Walia BN. Endoscopic sclerotherapy in children. J Gastroenterol Hepatol. (1989) 4(2):155–60. 10.1111/j.1440-1746.1989.tb00820.x 2490909

[8] Hill ID Bowie MD. Endoscopic sclerotherapy for control of bleeding varices in children. Am J Gastroenterol. (1991) 86(4):472–6. 2012050

[9] Oh SH Kim SJ Rhee KW Kim KM. Endoscopic cyanoacrylate injection for the treatment of gastric varices in children. World J Gastroenterol. (2015) 21(9):2719–24. 10.3748/wjg.v21.i9.2719 25759541 PMC4351223

[10] Shneider BL Bosch J de Franchis R Emre SH Groszmann RJ Ling SC Portal hypertension in children: expert pediatric opinion on the report of the Baveno V consensus workshop on methodology of diagnosis and therapy in portal hypertension. Pediatr Transplant. (2012) 16(5):426–37. 10.1111/j.1399-3046.2012.01652.x 22409296

[11] Stiegmann GV Goff JS Sun JH Davis D Silas D. Technique and early clinical results of endoscopic variceal ligation (EVL). Surg Endosc. (1989) 3(2):73–8. 10.1007/BF00590904 2788929

[12] Duché M Ducot B Ackermann O Guérin F Jacquemin E Bernard O. Portal hypertension in children: high-risk varices, primary prophylaxis and consequences of bleeding. J Hepatol. (2017) 66(2):320–7. 10.1016/j.jhep.2016.09.006 27663417

[13] Molleston JP Shneider BL. Preventing variceal bleeding in infants and children: is less more? Gastroenterology. (2013) 145(4):719–22. 10.1053/j.gastro.2013.08.026 23973849

[14] Ling SC. Advances in the evaluation and management of children with portal hypertension. Semin Liver Dis. (2012) 32(4):288–97. 10.1055/s-0032-1329897 23397529

[15] Galand J Ley D Coopman S Michaud L Guimber D Turck D Primary prophylaxis of oesophageal variceal bleeding in children by ligation is safe and as efficient as secondary prophylaxis. J Hepatol. (2018) 68(3):600–1. 10.1016/j.jhep.2017.07.040 28958884

[16] Saracco G Giordanino C Roberto N Ezio D Luca T Caronna S Fatal multiple systemic embolisms after injection of cyanoacrylate in bleeding gastric varices of a patient who was noncirrhotic but with idiopathic portal hypertension. Gastrointest Endosc. (2007) 65(2):345–7. 10.1016/j.gie.2006.07.009 17141231

[17] Burke MP O'Donnell C Baber Y. Death from pulmonary embolism of cyanoacrylate glue following gastric varix endoscopic injection. Forensic Sci Med Pathol. (2017) 13(1):82–5. 10.1007/s12024-016-9835-4 28091982

[18] Köksal AS Kayaçetin E Torun S Erkan V Ökten RS. Splenic infarction after N-butyl-2-cyanoacrylate injection for gastric varices: why does it happen? Surg Laparosc Endosc Percutan Tech. (2013) 23(5):e191–3. 10.1097/SLE.0b013e318272fd1e 24105294

[19] Belletrutti PJ Romagnuolo J Hilsden RJ Chen F Kaplan B Love J Endoscopic management of gastric varices: efficacy and outcomes of gluing with N-butyl-2-cyanoacrylate in a north American patient population. Can J Gastroenterol. (2008) 22(11):931–6. 10.1155/2008/389517 19018339 PMC2661196

[20] Sato T Yamazaki K. Evaluation of therapeutic effects and serious complications following endoscopic obliterative therapy with histoacryl. Clin Exp Gastroenterol. (2010) 3:91–5. 10.2147/CEG.S12189 21694852 PMC3108656

[21] Singer AD Fananapazir G Maufa F Narra S Ascher S. Pulmonary embolism following 2-octyl-cyanoacrylate/lipiodol injection for obliteration of gastric varices: an imaging perspective. J Radiol Case Rep. (2012) 6(2):17–22. 10.3941/jrcr.v6i2.845 22690282 PMC3370697

